# A novel small molecule CXCR4 antagonist potently mobilizes hematopoietic stem cells in mice and monkeys

**DOI:** 10.1186/s13287-020-02073-z

**Published:** 2021-01-07

**Authors:** Xiao Fang, Xiong Fang, Yujia Mao, Aaron Ciechanover, Yan Xu, Jing An, Ziwei Huang

**Affiliations:** 1 School of Life Sciences, Tsinghua University, Beijing, China; 2 The Rapport Faculty of Medicine, Technion-Israel Institute of Technology, 3109601 Haifa, Israel; 3 Nobel Institute of Biomedicine, Zhuhai, 519080 China; 4 Ciechanover Institute of Precision and Regenerative Medicine, School of Life and Health Sciences, Chinese University of Hong Kong, Shenzhen, China; 5 Division of Infectious Diseases and Global Public Health, Department of Medicine, School of Medicine, University of California at San Diego, La Jolla, CA USA

**Keywords:** Hematopoietic stem cell mobilization, CXCR4 antagonist, Monkeys, Hematopoietic stem cell transplantation

## Abstract

**Background:**

Hematopoietic stem cell (HSC) transplantation is an effective treatment strategy for many types of diseases. Peripheral blood (PB) is the most commonly used source of bone marrow (BM)-derived stem cells for current HSC transplantation. However, PB usually contains very few HSCs under normal conditions, as these cells are normally retained within the BM. This retention depends on the interaction between the CXC chemokine receptor 4 (CXCR4) expressed on the HSCs and its natural chemokine ligand, stromal cell-derived factor (SDF)-1α (also named CXCL12) present in the BM stromal microenvironment. In clinical practice, blocking this interaction with a CXCR4 antagonist can induce the rapid mobilization of HSCs from the BM into the PB.

**Methods:**

C3H/HEJ, DBA/2, CD45.1^+^, and CD45.2^+^ mice and monkeys were employed in colony-forming unit (CFU) assays, flow cytometry assays, and competitive/noncompetitive transplantation assays, to assess the short-term mobilization efficacy of HF51116 and the long-term repopulating (LTR) ability of HSCs. Kinetics of different blood cells and the concentration of HF51116 in PB were also explored by blood routine examinations and pharmacokinetic assays.

**Results:**

In this paper, we report that a novel small molecule CXCR4 antagonist, HF51116, which was designed and synthesized by our laboratory, can rapidly and potently mobilize HSCs from BM to PB in mice and monkeys. HF51116 not only mobilized HSCs when used alone but also synergized with the mobilizing effects of granulocyte colony-stimulating factor (G-CSF) after co-administration. Following mobilization by HF51116 and G-CSF, the long-term repopulating (LTR) and self-renewing HSCs were sufficiently engrafted in primary and secondary lethally irradiated mice and were able to rescue and support long-term mouse survival. In monkeys, HF51116 exhibited strong HSC mobilization activity and quickly reached the highest in vivo blood drug concentration.

**Conclusions:**

These results demonstrate that HF51116 is a new promising stem cell mobilizer which specifically targets CXCR4 and merits further preclinical and clinical studies.

## Background

CXC chemokine receptor 4 (CXCR4) [1] belongs to the superfamily of G-protein-coupled receptors (GPCRs) [2, 3] and has stromal cell-derived factor (SDF)-1α or CXCL12 as its natural ligand. The binding of hematopoietic stem cell (HSC)-expressing CXCR4 to microenvironmental SDF-1α causes transmission of signals to intracellular biological pathways [4, 5] that mediate many intracellular processes. The end result is HSC retention and proliferation in the hematopoietic organ bone marrow (BM) [6–8]. CXCR4 knockout mice show a severe deficiency in hematopoiesis [9], and this disruption of the SDF-1α/CXCR4 axis has contributed to the discovery and application of an effective HSC-mobilizing strategy [10–12].

HSCs are uncommitted cells and have the ability of self-renewal, differentiation into specialized hematopoietic cells, and reconstitution of the bone marrow. Traditionally, pre-transplant mobilization of HSCs was performed using granulocyte colony-stimulating factor (G-CSF) with or without chemotherapy [13, 14]. G-CSF can downregulate SDF-1α and promote HSC release to the PB [12, 15]. However, in order to collect sufficient quantity of HSCs, G-CSF-based mobilization requires multiple doses over a number of days, which is known to alter the function of the HSC niche, as well as bone formation, and can cause bone pain and spleen enlargement [16]. In addition, there is approximately 25% of failure rate in patients with the use of G-CSF with or without chemotherapy even when remobilizations are performed [17]. Inadequate or inter-individual variable numbers of HSCs can lead to delayed or failed engraftment, prolonged thrombocytopenia or neutropenia, increased infectious complications, and subsequently prolonged hospital stay or death [18–20]. These issues of G-CSF prompted the efforts to develop other HSC mobilization strategies based on targeted therapeutics. AMD3100 is a clinically approved CXCR4 antagonist used for HSC mobilization [21, 22]. When AMD3100 is used alone, approximately one-third of patients fail to mobilize the minimally acceptable amount of CD34-positive cells needed for allogeneic transplantation [23], and AMD3100 is often used in combination with G-CSF. In view of these, there is still the clinical need for new and effective therapeutics for HSC mobilization.

In the present study, we report the in vivo HSC mobilization efficacy of HF51116 [24], a novel CXCR4 antagonist developed recently by our laboratories. HF51116 possesses a very high CXCR4 binding affinity (IC_50_ = 12 nM) [25] and potently mobilizes HSCs from the bone marrow (BM) to the peripheral blood (PB). We have evaluated the efficacy of HF51116 in mice and monkeys, which demonstrates its potential as a new promising CXCR4 antagonist for clinical application in HSC transplantation.

## Methods

### Compound

Of the series of CXCR4 antagonists designed, we synthesized and identified a novel lead small molecule compound HF51116. The purity (> 98%) of HF51116 was checked by analytical high-performance liquid chromatography (HPLC), while the molecular weight (522.73 Da) and identity (C_29_H_46_N_8_O) of HF51116 were determined by high-resolution mass spectrometry (HRMS) and nuclear magnetic resonance (NMR).

### Mice

All mice were housed at the laboratory animal facility that had been accredited by AAALAC (Association for Assessment and Accreditation of Laboratory Animal Care International) and the IACUC (Institutional Animal Care and Use Committee) of Tsinghua University. Mouse animal protocols were approved by the laboratory animal facility. C57BL/6, C3H/HEJ, and DBA/2 mice were purchased from Charles River. We obtained B6.SJL-*Ptprc*^*a*^ *Pepc*^*b*^/BoyJ mice from Dr. Li Wu’s Lab (School of Life Science, Tsinghua University).

### Monkeys

Male rhesus macaques (4–6 years old) were housed in individual cages at the Institute of Laboratory Animals Science, Chinese Academy of Medical Sciences (CAMS) and Peking Union Medical College (PUMC), which had been accredited by AAALAC. The protocol was approved by the same institute with IACUC number XC19006.

### Colony-forming unit assay

The PB samples were obtained from mice and rhesus monkeys following injections of AMD3100, HF51116, and/or G-CSF. Ammonium chloride solution was used to remove the red blood cells. The remaining cells in suspension were cultured in MethoCult™ GF M3434 or MethoCult™ H4434 (STEMCELL Technologies) in a humidified atmosphere. The total numbers of colony-forming unit (CFU)-granulocyte-macrophage (CFU-GM), burst-forming unit-erythroid (BFU-E), and multipotential colony-forming unit-granulocyte, erythroid, megakaryocyte, and macrophage (CFU-GEMM) colonies were enumerated post 8–13 days of culture by the standard morphological criteria [26].

### Flow cytometry assay

Surface antigens were quantified by flow cytometry using ZE5 Cell Analyzer (Bio-Rad) and BD FACSAria™ III (BD). PE-labeled mouse anti-human CD34 [27], FITC-labeled mouse anti-mouse CD45.1, FITC-labeled mouse anti-NHP CD45, V450 mouse lineage antibody cocktail with isotype control, PE-labeled mouse anti-mouse CD45.2, FITC-labeled hamster anti-mouse CD48, PE-Cy™7-labeled rat anti-mouse Ly-6A/E, APC-labeled rat anti-mouse CD117 (BD Biosciences), and PE-labeled anti-mouse CD150 (SLAM) (BioLegend) antibodies were used in the flow cytometry assays.

### Long-term repopulating assay

The F1 generation (CD45.1/CD45.2) of the C57BL/6 and B6.SJL-*Ptprc*^*a*^ *Pepc*^*b*^/BoyJ crosses served as recipients. In the first repopulation competitive assay, G-SCF (100 μg/kg, every 12 h for 4 days; Ohtemachi, Chiyoda-ku, Tokyo, Japan) was subcutaneously injected into CD45.2^+^ mice. At 12 h post-final G-CSF injection, saline, 5 mg/kg HF51116, or 5 mg/kg AMD3100 (C-aring, Wuhan, China) was subcutaneously injected. WBCs were isolated immediately, 30 min (min), and 1 h after injection in each group. CD45.1^+^ BM cells were also isolated. The competitor cell number (CD45.1^+^ cells) was 0.5 × 10^6^, and the donor cell number (CD45.2^+^ cells) was 1.0 × 10^6^. Cell suspension, 1.5 × 10^6^ cells, was intravenously injected into lethally irradiated CD45.1/CD45.2 mice (11 Gy, 5.5 Gy split dose, 2 h apart, radiation rate 1.05 Gy/min). The percentages of CD45.2^+^ cells were checked every month for 6 months. At that time, the noncompetitive assay was performed to determine the secondary repopulation. Briefly, lethally irradiated CD45.1/CD45.2 mice received the BM cells from each group of recipients. The percentages of CD45.2^+^ cells were also checked.

### Pharmacokinetic assays

HF51116 was s.c. injected into rhesus monkeys at 1 and 10 mg/kg. The concentration of HF51116 in serum was checked by LC-MS (Thermo Fisher, CA). ACQUITY UPLC BEH C18 column (2.1 × 100 mm, 1.7 μm, Waters) was used to separate the extracts. The binary solvent system included mobile phase A (0.1% formic acid and 5 mM ammonium acetate in 100% H_2_O) and mobile phase B (100% acetonitrile). A 10-min gradient with 250 μL/min flow rate was used as follows: 0–1.5 min, 2% B; 1.5–5 min, 2–98% B; 5–7 min, 98% B; 7–7.1 min, 2% B; and 7.1–10 min, 2% B. Data acquired in selected reaction monitoring (SRM) for HF51116 with transitions of 523.5/161.

### Blood routine examination

All blood samples underwent blood routine examination using a ProCyte Dx Hematology Analyzer (IDEXX).

### Statistical analysis

Prism (GraphPad) and Xcalibur (Thermo Fisher, CA) were used for one-way ANOVA, two-way ANOVA analysis, and descriptive statistics. Data were shown as mean ± SEM. The flow cytometry data were processed by FlowJo (FLOWJO).

## Results

### Mobilization of different peripheral blood cells in mice

**Fig. 1:**
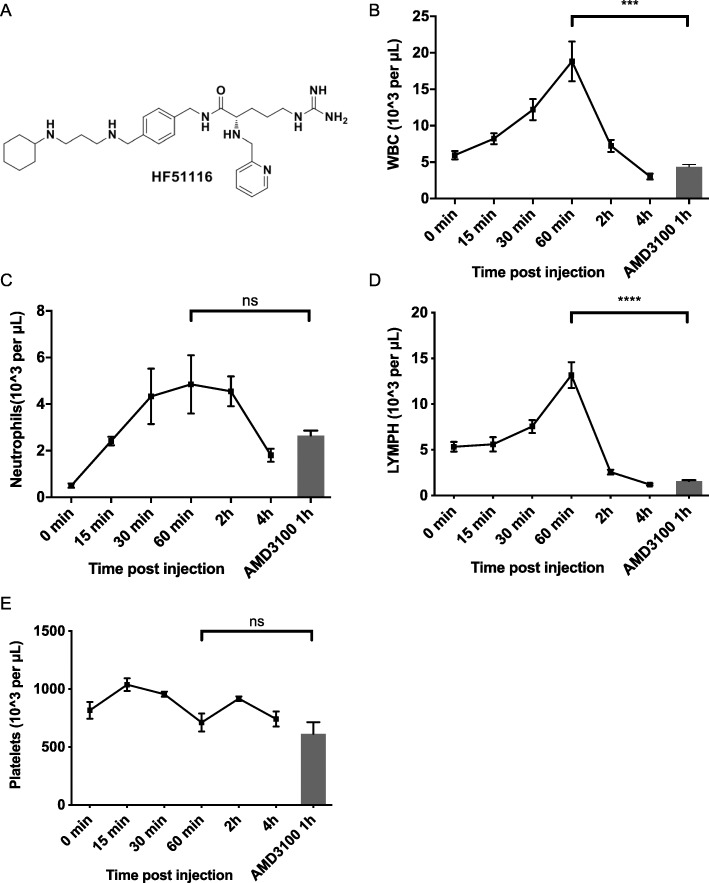
HFX51116 induced kinetic changes of different blood cells in mice*.* **a** The chemical structure of HF51116. Five milligrams per kilogram HF51116 or 5 mg/kg AMD3100 was subcutaneously injected into C57BL/6 mice. At different times post-injection, dynamic changes of WBCs (**b**), neutrophils (**c**), lymphocytes (**d**), and platelets (**e**) in PB were tested (mean ± SEM of *n* = 6 mice/time point)

Through our extensive research efforts over many years, we have developed a new class of small molecule agents that are potent antagonists of CXCR4. On the basis of our representative compound HF50731 [28], we have developed a new highly potent small molecule analog named HF51116, which features an unsymmetrical polyamine (Fig. 1a). HF51116 binds strongly to CXCR4 with the IC_50_ of 12 nM in competitive binding with 12G5 [25]. We examined the compositions and dynamics of different PB cells in mice following subcutaneous injection of HF51116. The PB showed time-dependent changes in WBCs and neutrophils in response to HF51116. At 5 mg/kg, total WBC numbers in PB achieved a maximum number (18.83 K/μL) at 60 min post HF51116 injection (Fig. 1b). Increases in neutrophil numbers occurred faster and lasted longer when compared to 0 min (0.50 K/μL), with increases of approximately 9-fold occurring from 30 min to 2 h after HF51116 treatment and 3.6-fold increases observed at 4 h (Fig. 1c). The lymphocyte numbers (Fig. 1d) started to increase at 30 to 60 min, followed by a dramatic decrease at 1 to 4 h. At the same dose and time post-injection of HF51116 and AMD3100, HF51116 escalated more WBCs and lymphocytes in PB (Fig. 1b, d). No changes were observed in platelet numbers in the PB in response to HF51116 injection, when compared to 0 min (Fig. 1e, 816.83 K/μL), suggesting that HF51116 specifically mobilized WBCs.

### Rapid mobilization of hematopoietic progenitor cells (HPCs) in mice

**Fig. 2:**
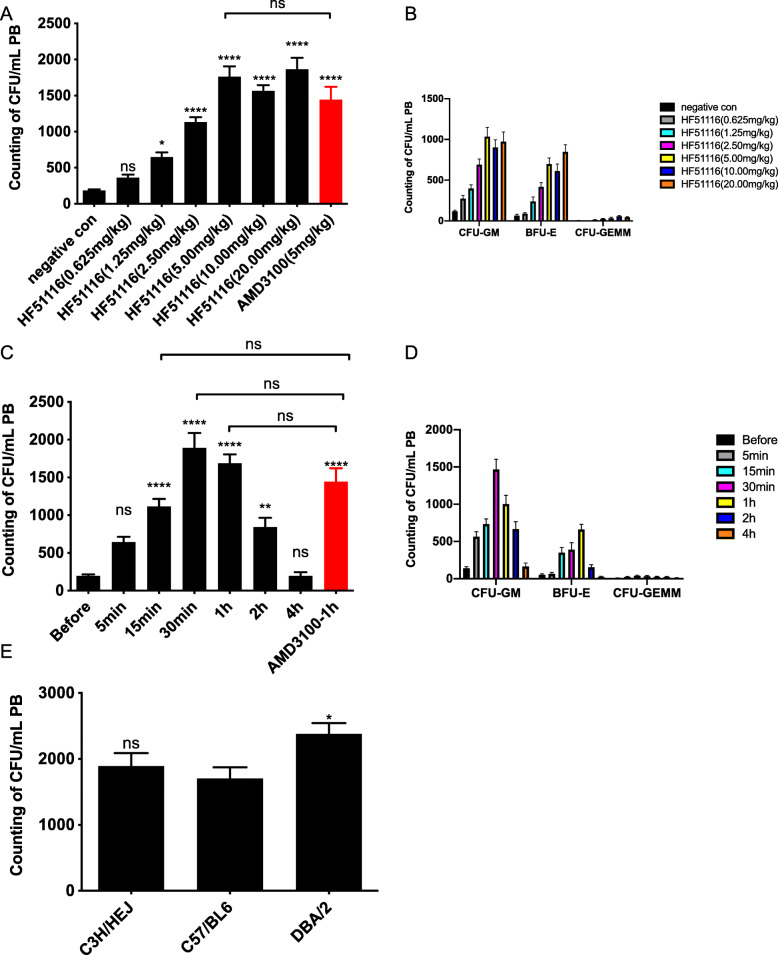
Mobilization of murine HPCs by HF51116. **a**, **b** The dose-dependent response of HF51116 in C3H/HEJ mice. HF51116 was given at different doses and AMD3100 was given at 5 mg/kg. The negative control group received saline only. Blood samples were collected at 1 h post-injection. **a** Sum of CFUs at different doses. **b** Composition of CFUs at different doses (mean ± SEM of *n* = 12 mice/group/dose). **c**, **d** Dynamic change of efficacy at different times following subcutaneous (s.c.) injections of HF51116 (5 mg/kg) or AMD3100 (5 mg/kg) into C3H/HEJ mice. HF51116 and AMD3100 were given at the same dose. CFU numbers were measured at different times for HF51116 and at 1 h for AMD3100. **c** Sum of CFUs at different times. **d** Composition of CFUs at different times (mean ± SEM of *n* = 10 mice/time point, *n* = 9 mice for 4 h, *n* = 12 mice for the AMD3100 group). **e** Inter-individual variability in different mouse strains. Five milligrams per kilogram HF51116 was subcutaneously (s.c.) injected into C57BL/6, C3H/HEJ, and DBA/2 mice. The blood samples were collected at 30 min post-injection (mean ± SEM of *n* = 12 mice for the C57BL/6 and DBA/2 groups, *n* = 10 mice for the C3H/HEJ group). ****P < 0.0001, **P < 0.01, *P < 0.05; ns, not significant

We demonstrated that the HPC mobilization induced by HF51116 was dose and time dependent (Fig. 2a–d). At 1 h post-injection of HF51116, the colony numbers reached a plateau at 5 mg/kg, with no further increase at 10 mg/kg and 20 mg/kg (Fig. 2a). The plateau level was about 9.36-fold higher than the baseline circulating level (Fig. 2a, the negative control group, 185 CFUs/mL). The mobilizing efficacy of HF51116 was comparable to AMD3100 [29] at a dose of 5 mg/kg. In comparison with the negative control group, HF51116 induced 8.73-fold increases in CFU-GM (1035/mL), 11.01-fold increases in BFU-E (698/mL), and 9.75-fold increases in CFU-GEMM (33/mL) numbers in PB (Fig. 2b).

CFUs were also rapidly increased at 15 min after HF51116 treatment and reached a pick level at 30 min, 9.57-fold higher than the level in PB collected before HF51116 injection (Fig. 2c, the Before group, 198 CFUs/mL). The mobilization efficacy of HF51116 was comparable to that of AMD3100 from 15 min to 1 h post-injection (Fig. 2c). The PB also showed time-dependent changes in CFU-GMs, BFU-Es, and CFU-GEMMs in response to HF51116 (Fig. 2d).

Inter-individual variability in the mobilizations of HPCs in patients means that approximately 15% of patients are insensitive to G-CSF [18]. This phenomenon also exists in different mouse strains [9]. We used C57BL/6, C3H/HEJ, and DBA/2 mice to test the variability in HF51116 response and sensitivity to HF51116 in different mouse strains (Fig. 2e). At 30 min post-injection of 5 mg/kg HF51116, C57BL/6 and C3H/HEJ strains showed comparable sensitivities, but DBA/2 strain exhibited the better sensitivity than C57BL/6 strain.

### Synergistic mobilization by HF51116 and G-CSF

After confirming the optimal dose and time for HPC mobilization, we tested the potential for synergistic effects of co-administration efficiency of G-CSF+HF51116.

**Fig. 3:**
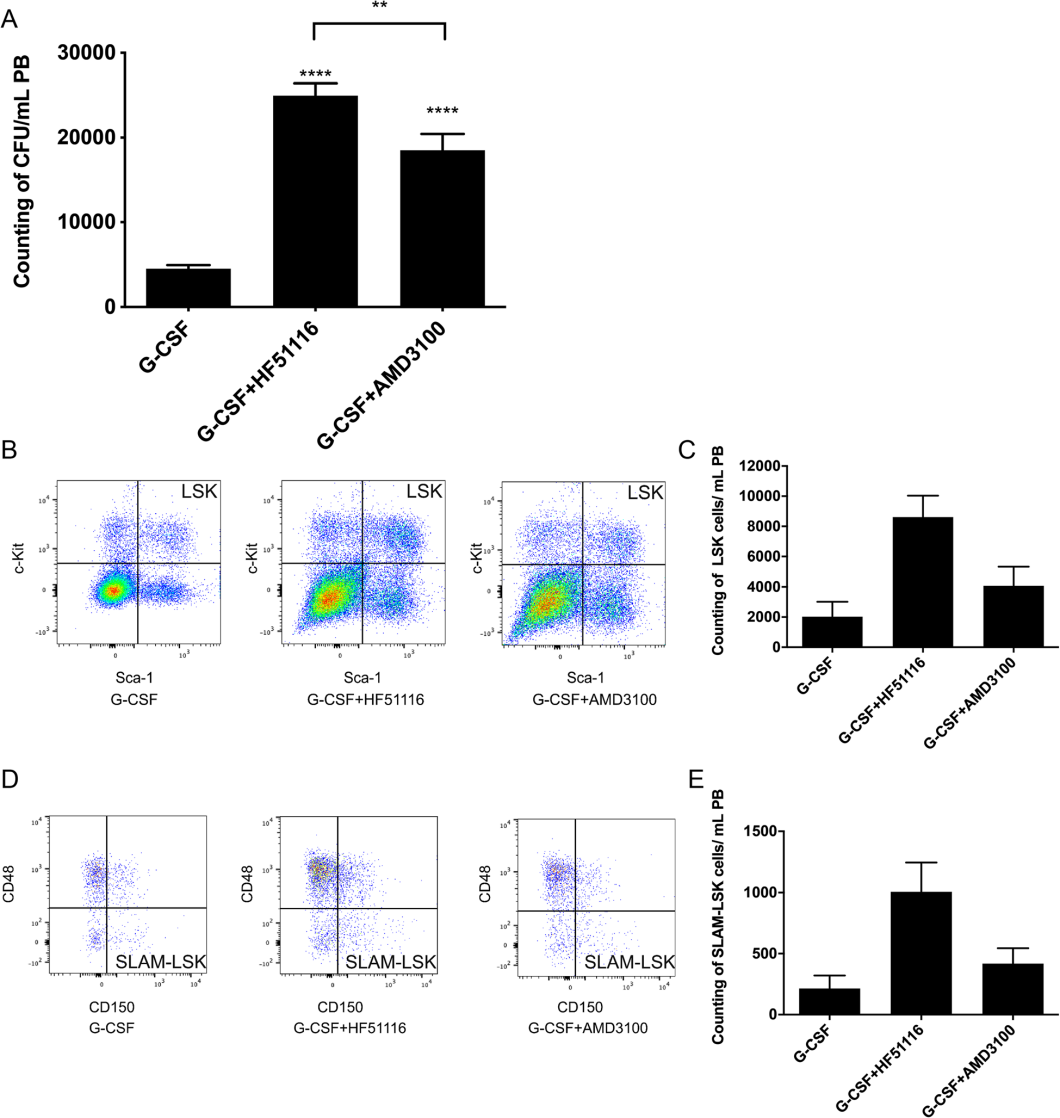
Synergistic mobilization by HF51116 and G-CSF. **a** One hundred micrograms per kilogram G-CSF was subcutaneously injected (s.c.) into C57BL/6 mice every 12 h for 4 days. Saline, 5 mg/kg HF51116, or 5 mg/kg AMD3100 was subcutaneously injected (s.c.) at 12 h post-final G-CSF injection. Total CFU numbers were tested at 0, 30, and 60 min post-injection (mean ± SEM of *n* = 16 mice). ****P < 0.0001, ***P < 0.001, **P < 0.01; ns, not significant. **b**–**e** The absolute number of LSK and SLAM-LSK cells in peripheral blood post different treatments. The treatment regimen was the same as described in **a**. Counting of **b**, **c** HSPCs (LSK: Lineage^−^ Sca-1^+^ c-Kit^+^) and **d**, **e** HSCs (SLAM LSK: lineage^−^ Sca-1^+^ c-Kit^+^ CD150^+^ CD48^−^) were analyzed through flow cytometry (mean ± SEM of *n* = 3 mice for the G-CSF and G-CSF+AMD3100 group, *n* = 4 mice for the G-CSF+HF51116 group)

G-CSF, G-CSF+HF51116, or G-CSF+AMD3100 were subcutaneously injected into mice. The mobilization efficacy in the G-CSF+HF51116 group (24,963 CFUs/mL) was 5.50-fold higher than that in the G-CSF group (4538 CFUs/mL) and 1.35-fold higher than that in G-CSF+AMD3100 group (18,512 CFUs/mL) (Fig. 3a). We simultaneously examined the absolute number of hematopoietic stem and progenitor cells (HSPCs, Lineage^−^ Sca-1^+^ c-Kit^+^: LSK) and HSCs (CD150^+^ CD48^−^ lineage^−^ Sca-1^+^ c-Kit^+^: SLAM LSK) in PB mobilized by these three treatments [30]. The absolute number of LSK cells in the G-CSF+HF51116 group and G-CSF group were 8612/mL and 2024/mL, respectively (Fig. 3b, c). In addition, the absolute number of SLAM-LSK cells in the G-CSF+HF51116 group and G-CSF group were 715/mL and 214/mL, respectively (Fig. 3d, e). A tendency to a higher absolute number of LSK and SLAM-LSK cells in the G-CSF+HF51116 group was observed.

### Long-term repopulating and self-renewing capability of HSPCs and HSCs mobilized by G-CSF and HF51116

**Fig. 4:**
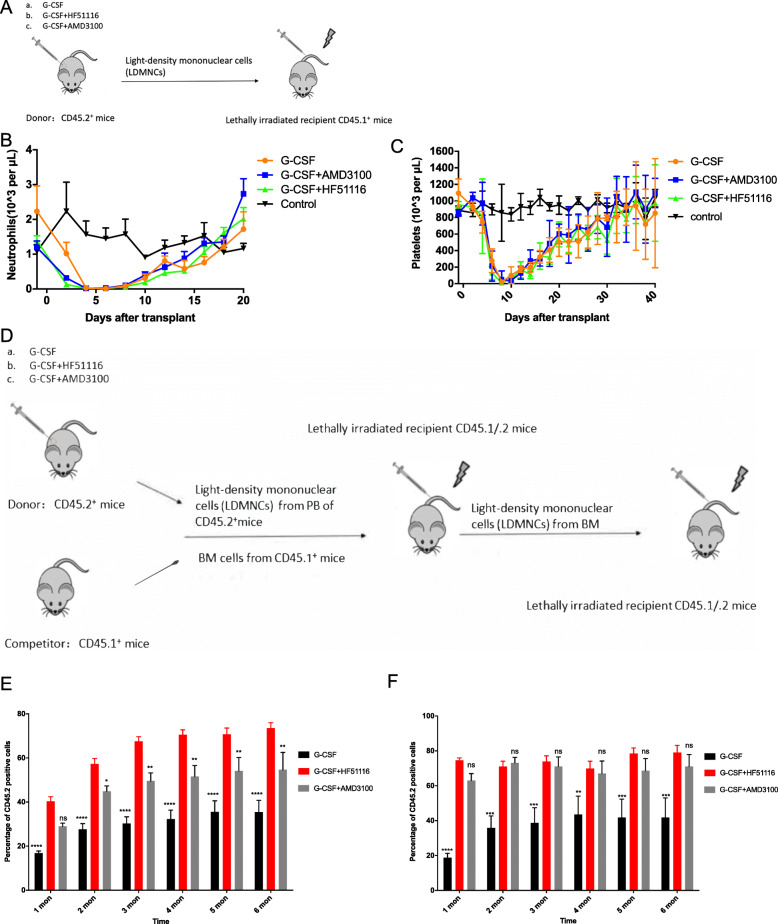
Engraftment kinetics and long-term repopulating capability of HPCs mobilized by HF51116+G-CSF. **a** Early engraftment strategy. G-SCF (100 μg/kg, every 12 h for 4 days) was subcutaneously injected into CD45.2^+^ mice. At 12 h post-final G-CSF injection, saline, 5 mg/kg HF51116, or 5 mg/kg AMD3100 was subcutaneously injected into the CD45.2^+^ mice. Light-density mononuclear cells (LDMNCs) were collected in PB at 0, 30, and 60 min post-injection of each of these agents. Lethally irradiated CD45.1^+^ recipients received a graft of LDMNCs. The control group was healthy mice with no radiation. The recoveries of neutrophils (**b**) and platelets (**c**) were monitored every 2 days for 40 days (mean ± SEM of *n* = 10 mice/group). **d** Competitive repopulation assay strategy using CD45 congenic mice. G-CSF, G-CSF+HF51116, or G-CSF+AMD3100 were injected into CD45.2 mice. Drug administration strategy was the same as used in **a**. BM cells from CD45.1 mice and LDMNCs from CD45.2 mice were isolated. The competitor cell number (CD45.1^+^ cells) was 0.5 × 10^6^, and the donor cell number (CD45.2^+^ cells) was 1.0 × 10^6^. Cell suspension containing donor and competitor cells (1.5 × 10^6^ cells) was intravenously injected into lethally irradiated (11 Gy, 5.5 Gy split dose, 2 h apart, radiation rate 1.05 Gy/min) CD45.1/CD45.2 recipients. **e** The percentages of CD45.2^+^ cells were checked for 6 months (mean ± SEM of *n* = 7 mice). **f** The secondary repopulation in a noncompetitive assay. At 6 months post-injection, lethally secondary irradiated CD45.1/CD45.2 mice received the BM cells of every group of recipients (**e**) in a noncompetitive assay. The percentage of CD45.2^+^ was checked every month (mean ± SEM of *n* = 7 mice). ****P < 0.0001, ***P < 0.001, **P < 0.01, *P < 0.05; ns, not significant

We also evaluated the long-term repopulating and self-renewing ability of HSPCs and HSCs post-injection of G-CSF+HF51116. The recovery of neutrophils and platelets was reflected in the engraftment kinetics, as the HSPCs and HSCs mobilized by G-CSF+HF51116 treatment showed timely and early engraftment (Fig. 4a–c). Lethally irradiated CD45.1^+^ recipients receiving light-density mononuclear cells (LDMNCs) obtained from PB mobilized by G-CSF, G-CSF+AMD3100, and G-CSF+HF51116 (Fig. 4a) showed similar engraftment kinetics: neutrophils recovered to the baseline level at around 18 days (Fig. 4b) and platelets recovered at around 35 days (Fig. 4c).

We employed CD45 congenic mice to demonstrate the long-term repopulating ability of HSPCs and HSCs (Fig. 4d). The percentage of CD45.2^+^ cells in the G-CSF+HF51116 (73.50%) group was 2.0-fold higher than in the G-CSF group (35.44%) and 1.3-fold higher than in the G-CSF+AMD3100 (54.60%) group post 6 months transplantation (Fig. 4e). We also collected CD45.1/CD45.2 mouse BM cells and tested self-renewal of the long-term repopulated cells in a noncompetitive pattern of secondary transplantation (Fig. 4d). There was no significant difference of the percentage of CD45.2^+^ cells between G-CSF+HF51116 (79.11%) and G-CSF+AMD3100 (70.98%) groups; the percentage of CD45.2^+^ cells was still 2.0-fold higher in the G-CSF+HF51116 group than in the G-CSF group (41.76%) after 6 months post-transplantation (Fig. 4f). These data confirmed that the HSPCs and HSCs mobilized by G-CSF+HF51116 not only produce timely and early engraftment but they also retain a long-term repopulating and self-renewing capability.

### Mobilization of different peripheral blood cells in monkeys

**Fig. 5:**
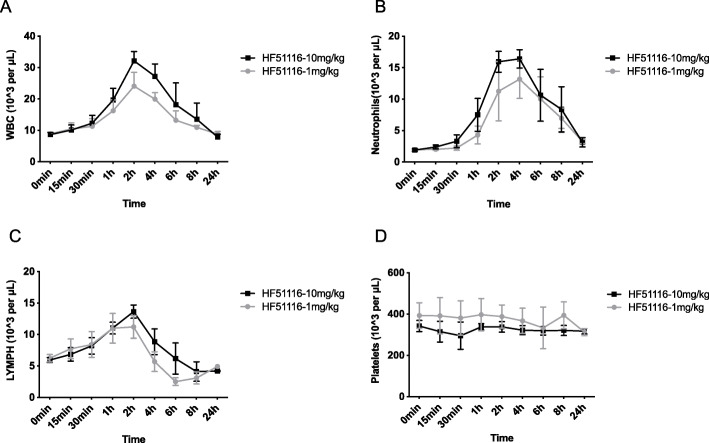
Kinetics of different blood cells mobilized by HF51116 in monkeys. The dynamic changes of WBCs (**a**), neutrophils (**b**), lymphocytes (**c**), and platelets (**d**) in peripheral blood following subcutaneously injections of 10 or 1 mg/kg HF51116 into rhesus monkeys. Blood samples were collected at different times post-injection (mean ± SEM of *n* = 3 rhesus monkeys/group/time point)

We addressed mobilization activity of HF51116 in monkeys [31]. HF51116 was subcutaneously injected into rhesus monkeys at 10 or 1 mg/kg. Kinetics of WBCs, neutrophils, and lymphocytes were in time-dependent manners (Fig. 5a–c). A maximum number of WBCs was achieved at 2 h (Fig. 5a). HF51116 induced a 3.73-fold (10 mg/kg) change in WBC numbers when compared to 0 min (average 8.87 K/μL) with the *P* value of 0.0015. Neutrophil numbers reached the highest level at 4 h post-injection of 10 mg/kg and 1 mg/kg of HF51116 (Fig. 5b). Lymphocytes showed the maximum increases in number at 2 h for both doses (Fig. 5c), similar to the WBC response. However, lymphocyte numbers decreased quickly from 2 to 8 h and had reached 70% of the 0-min value by 24 h. HF51116 did not induce changes in platelets when compared to 0 min at either dose, similar to the change in mouse (Figs. 5d and 1d).

### Mobilization of HSCs in monkeys

**Fig. 6:**
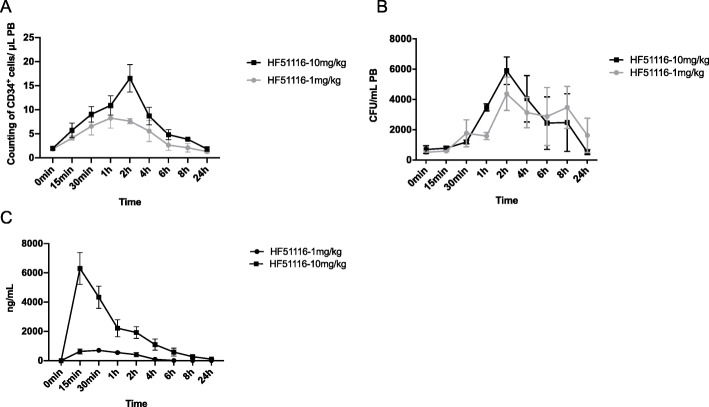
Mobilization efficacy and PK properties of HF51116 in monkeys. HF51116 at 10 and 1 mg/kg were subcutaneously injected into rhesus monkeys. Blood samples were collected at different times post-injection. **a**, **b** Time response of CD34^+^ cells and HPCs in monkeys. **c** Concentration changes of HF51116 in PB (mean ± SEM of *n* = 3 rhesus monkeys/group/time point)

We examined the CD34^+^ cell counts [32, 33] in the PB in monkeys (Fig. 6a) [34] and determined that the HF51116-induced HPC mobilization was time dependent (Fig. 6b). Two hours post-injection of 10 mg/kg HF51116, there were 17 CD34^+^ cells/μL PB. The area under the curve (AUC) was 38.47 for dose 1 mg/kg and 61.50 for dose 10 mg/kg (Fig. 6a). At 2 h post-injection, 10 (5900 CFUs/mL) or 1 mg/kg (4373 CFUs/mL) HF51116 produced maximum HPC mobilization effects at the same time point when WBCs and CD34^+^ cells reached their maximum numbers. One milligram per kilogram HF51116 induced an approximately 8.5-fold increase when compared to 0 min (510 CFUs/mL) with the *P* value of 0.0251 (Fig. 6b).

At 10 mg/kg, the highest concentration of HF51116 (6305.89 ng/mL) in the PB plasma occurred at 15 min post-injection (Fig. 6c), and 100.54 ng/mL remained in the plasma at 24 h. At 1 mg/kg, the highest concentration (704.641 ng/mL) of HF51116 appeared at 30 min and no HF51116 remained in the plasma 8 h later.

## Discussion

Blocking the SDF-1α/CXCR4 axis can elicit rapid mobilization of HSCs from the BM to the PB in clinical practice [35], as demonstrated by the clinically approved CXCR4 antagonist AMD3100 [29]. Our recent efforts in developing new CXCR4 antagonists led to the discovery of HF51116 [25], a novel small molecule which strongly and specifically binds CXCR4 and effectively blocks SDF-1α-induced CXCR4^+^ cell migration and calcium influx (unpublished results).

For in vivo efficacy, we assessed the dose-dependent and time-dependent responses in C3H/HEJ mice and the mobilization effect in three different mouse strains. DBA/2 mice were more sensitive than C57BL/6 mice to HF51116. This variation resembles the situation of inter-individual variability in human patients and in mice in response to POL5551 [36]. Our investigation of the in vivo efficacy for mobilizing HPCs in both mice and monkeys confirmed that HF51116 consistently and significantly mobilized CFUs from the BM to the PB. The HSC mobilization efficacy of HF51116 and AMD3100 was compared in mice. The HF51116-induced mobilization of HPCs was dose and time dependent. At 5 mg/kg, the number of CFUs mobilized at 15 min post-injection of HF51116 was the same as that achieved at 1 h post-injection of AMD3100. Therefore, HF51116 might be a potential alternative to AMD3100 for patients who do not respond to AMD3100. In monkeys, the maximum CD34^+^ cell and CFU mobilization effects also occurred at 2 h. The kinetics of WBC, neutrophil, and lymphocyte mobilization by HF51116 were also time and dose dependent. HF51116 could induce the increase of neutrophils in PB for a long time in mice and monkeys. On the other hand, the numbers of platelets in PB remained unchanged in mice and monkeys after HF51116 injection. HF51116 reached the highest blood concentration at 15 min post-injection and was subsequently removed from the blood circulation quickly in monkeys.

The repopulating activity was studied by examining the effect of HF51116 on the long-term repopulation in CD45 congenic mice. HF51116 dramatically synergized the mobilizing capacity of G-CSF after co-administered into these mice. In noncompetitive assays, HSCs and HSPCs mobilized by HF51116+G-CSF provided timely engraftment. In competitive assays, the percentage of CD45.2^+^ cells was higher in the HF51116+G-CSF group than in the G-CSF and AMD3100+G-CSF groups in the primary transplant. Treatment by G-CSF may either elicit inflammatory signals or promote HSC proliferation to increase the mobilizable HSC pools. HF51116 acts on the SDF-1α/CXCR4 axis and mobilizes more HSCs than using G-CSF alone. G-CSF shows inter-individual variability and causes bone pain due to its toxic side effects on the BM microenvironment [37, 38], and it requires a 4-day standard treatment, which could be too long. The stronger HSC mobilization activity of HF51116 plus G-CSF was clearly demonstrated in the primary transplant and the activity was similar with AMD3100 plus G-CSF in the secondary transplant. This short-term higher HSC mobilization activity of HF51116 plus G-CSF might represent a possible option for patients who suffer from the side effects of chemotherapy. In addition, the combination of HF51116 with other drugs such as proteasome inhibitors [39, 40], GRO β [34, 41, 42], or Viagra [43] is worth investigating to find even better and shorter HSC mobilization regimens.

## Conclusions

In summary, we have shown that a novel CXCR4 antagonist HF51116 is a potent HSC mobilizer and can rapidly and sufficiently mobilize HSCs from BM to the PB in mice and monkeys. HF51116 not only mobilizes HSCs when used alone, but also synergizes with G-CSF when co-administered. The HSCs mobilized by HF51116 have long-term repopulating activity and are sufficient for engrafting in primary and secondary lethally irradiated mice, where they rescue and support the survival of the animals. In monkeys, HF51116 exhibits strong HPC mobilization activity and is removed from the circulation quickly. These results demonstrate that HF51116 represents a novel and potent HSC mobilizer that targets CXCR4 and has promising potential for clinical applications.

## Data Availability

The data and materials are available from the corresponding authors upon request.

## Abbreviations

BM: Bone marrow
BMSCs: Bone marrow stromal cells
BFU-E: Burst-forming unit-erythroid
CFU-GEMM: Colony-forming unit-granulocyte, erythroid, megakaryocyte, and macrophage
CFU-GM: Colony-forming unit-granulocyte-macrophage
CXCR4: CXC chemokine receptor 4
G-CSF: Granulocyte colony-stimulating factor
HSPC: Hematopoietic stem and progenitor cell
HSCs: Hematopoietic stem cells
HSCT: Hematopoietic stem cell transplantation
LTR: Long-term repopulating
PB: Peripheral blood
SDF-1α: Stromal cell-derived factor-1α
WBCs: White blood cells
